# The Effect of Capital Structure on the Profitability of Pharmaceutical Companies The Case of Iran

**Published:** 2013

**Authors:** Mehdi Mohammadzadeh, Farimah Rahimi, Forough Rahimi, Seyed Mohammad Aarabi, Jamshid Salamzadeh

**Affiliations:** a *School of Pharmacy, Shahid Beheshti University of Medical Sciences, Tehran, Iran.*; b *Tarbiat Modares University, Management, Tehran, Iran.*; c *Facalty of Pharmacy, Tehran University of Medical Sciences, Tehran, Iran.*

**Keywords:** Capital Structure, Profitability, Pharmaceutical industry, Iran

## Abstract

Funding combination is the most important issue for the companies while they know the amount of required capital. Companies should be careful regarding the appliance of financial providing methods compatible with the investment strategy of company and profitability. This study seeks to examine the relationship between the capital structure and the profitability of pharmaceutical companies in Iran. For this purpose, top 30 Iranian pharmaceutical companies defined as study samples and their financial data were gathered for the period of 2001-2010. In this study, the net margin profit and debts to asset ratio were used as indicators of profitability and capital structure, respectively and sales growth was used as a control variable. Results showed that there was significant negative relationship between the profitability and the capital structure which means that the pharmaceutical companies have established a Pecking Order Theory and the internal financing has led to more profitability.

## Introduction

The total Volume of Iran pharmaceutical Market was around 4 billion dollars in 2011, which embodies the 15% growth annually during 5 last years. 65% of this market needs is provided by domestic producers and 35% by multinational companies from other countries. (MOH, annual report) As pharmaceutical industry is quite a lucrative and promising one in Iran, it is worth noting the factors influencing this industry and perusing its profitability; factors such as decisions over investing, and financing, which have a close relationship with foresight in any organization.

In the process of investing, organizations normally trade off the present benefits for the future benefits and use the present finances to be able to fulfill their own commitments to the financial suppliers in the future.

The choice of financing reflects the trade-off between the tax benefits of debt and associated bankruptcy and agency costs. Company’s capital structure largely depends on company-specific factors such as the probability of bankruptcy, profitability, quality and structure of assets. Beyond these factors, company’s industry affiliation and characteristics of country the company operates also influence financing structure. Thus, choice of the capital structure is an individual decision of each company (1).

Creating optimal capital structure, that is determining the most beneficial proportions of equity and borrowed financing in the capital structure, is one of the main tasks for the process of financial management (2).

Harris and Raviv (1991), Maksimovic and Zechner (1991), Chevalier(1995), Kovenoch and Philips (1995), and Mackey and Philips (2001) have demonstrated that the industry type affects the use of debt and in general, the company’s overall performance. Therefore, to test our theory, in this study, we have surveyed the relationship between the capital structure and profitability among the pharmaceutical companies present in the Tehran Stock Exchange (TSE).

Profitability is a factor centering on the two theories of “Static Trade-off Theory” and “Pecking Order Theory” on the capital structure. 

The trade off theory says that firms seek debt levels that balance the tax advantages of additional debt against the costs of possible financial distress. The tradeoff theory predicts moderate borrowing by tax-paying firms. The pecking order theory says that the firm will borrow, rather than issuing equity, when internal cash flow is not sufficient to fund capital expenditures. Thus the amount of debt will reflect the firm’s cumulative need for external funds. (3).

Due to the interconnectivity of capital structure and the profitability, the company managers put all their efforts on reaching a suitable form of the combination between financial resources and the proper capital structure.

Around half a century ago, Weston (1955) initiated the discussion over the possibility of registering the theories of combining financial resources; and the follow-up of such issue led to the first capital structure theory by Modigliani and Miller (1958). In 1963, Modigliani and Miller edited their initial proposed theories and modified their theory in the following way: Due to the tax benefit caused through borrowing, borrowing is a good choice for the financial supplement as borrowing leads to an increase in the company value.

**Figure 1:**
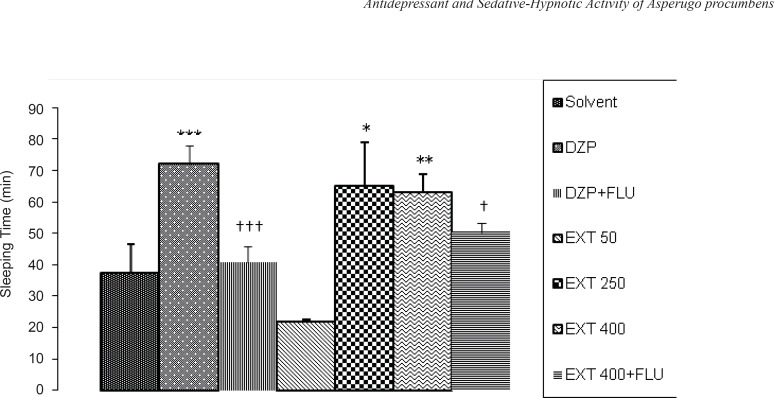
Trend of net income in Iranian Pharmaceutical Companies

Generally, all of the done researches in this area can be divided into 3 groups: Group 1: Mayers (1977), Miller (1977), Dammon and Senbet (1988), Fernandez (2001), and Hovakimian (2001): They reached to a significant and positive relationship between the return of equity and liabilities; which means, the more liabilities cause more return of equity. Hadlock and James (2002) by studying 500 companies between the years of 1980 to 1993 have shown that more profitable companies have used borrowed financial resources more than equity, on the other word the main financial resources of such companies were in debt. Group 2: Friend & Lang (1988) have studied 948 American companies during 1979 up to 1983. They explained that there is a significant negative relationship between the debt and profitability in these companies. Fama & French (1998) also concluded that debt never leads to access to the tax advantages. On the other hand, more borrowing leads to conflict of interest between managers and owners (agency theory) that it can create negative relationship between profitability and long term debt ratio finally. Group 3: Lara and Mesquita (2003) reached to a positive relationship between profitability , short-term debt ratio and equity; but they also found a negative relationship between profitability and long-term debt ratio through a study of 70 Brazilian companies during 1995 to 2001. Abor (2005) through a study over companies in Ghana during 1998 to 2002, concluded that there is a positive relationship between the total debt to asset ratio and profitability, and positive tie between short-term debt to asset ratio and profitability; they also showed a negative relationship between long-term debt to asset ratio and profitability.

## Experimental

To study the relationship between capital structure and profitability in pharmaceutical companies, we selected all listed pharmaceutical companies in TSE from 2001 till 2010 (by considering the two conditions: existence of the needed variables and presence in TSE in this period). Therefore, financial information of 26 pharmaceutical companies which were active in TSE qualified in this stage were derived out of the balance sheets and income statements (These companies supply around 80% of domestic produced medicines).

The variables used in this study are divided into three groups. The first is related to the capital structure including: the Debt to Total Asset ratio (TD/TA), short-term Debt to Total Asset ratio (ST/TA), and the long-term Debt to Total Asset ratio (LD/TA).

The second group variables show the profitability of the companies: the net profit margin (NPM), Return on Asset (ROA) and Return on Equity (ROE). The company size (SF) variable has been used as the control variable and is placed in the 3^rd ^group. We used descriptive statistics to analyze and describe the data (Table 1).

**Table 1:** Descriptive statistics on data

	**SD/TA**	**LD/TA**	**TD/TA**	**NPM**	**ROA**	**ROE**	**SF**
**Mean**	0.689	0.030	0.719	0.292	0.215	0.827	5.349
**Median**	0.692	0.031	0.720	0.290	0.206	0.730	5.324
**Maximum**	0.804	0.036	0.833	0.372	0.282	1.231	5.642
**Minimum**	0.531	0.023	0.559	0.203	0.170	0.574	5.087
**.Std. Dev**	0.077	0.004	0.079	0.052	0.032	0.236	0.168
**Skewness**	-0.480	-0.507	-0.465	0.013	0.878	0.678	0.327
**Kurtosis**	2.493	1.907	2.427	2.234	2.878	1.795	2.195
**Jarque-Bera**	12.769	24.100	12.922	6.371	33.583	35.653	11.654
**Probability**	0.002	0.000	0.002	0.041	0.000	0.000	0.003
**Sum**	179.062	7.904	186.966	75.825	55.894	215.018	1390.638
**.Sum Sq. Dev**	1.555	0.005	1.609	0.708	0.266	14.478	7.287
**Observations**	300	300	300	300	300	300	300
**Cross sections**	30	30	30	30	30	30	30

In the following graphs, net income of the companies present in this period of stock market has been diagramed. To get the profit to a fixed price, the producer’s price (2004 = 100), chemical production index, and the chemical products presented by the Central Bank of Iran (CBI) has been used for the purpose of adjustment. To investigate the connection between capital structure and the profitability of these pharmaceutical companies, we use the correlation coefficient for the purpose of finding the most relevant variables. We used SPSS to assess the correlation between capital structure and profitability ratio (net profit margin, Return on Asset and Return on Equity).

As can be seen in Table 2, from among the variables considered for profitability, sale revenue has more correlation with the capital structure variables. Therefore, we used the profit ratio to the sale as the profitability variable.

**Table 2:** Correlation coefficient between the variables of capital structure and profitability

**Capital structure variables**	**short-term debt to the net asset (SD/TA)**	**long-term debt to the net asset (LD/TA)**	**Debt to the total capital (TD/TA)**
**Profitability Variables**			
**NPM**	R= -0/488 p-value= 0/00	R= -0/025 p-value= 0/687	R= -0/480 p-value= 0/00
**ROA**	R= -0/369 p-value= 0/00	R= -0/105 p-value= 0/091	R= -0/391 p-value= 0/00
**ROE**	R= 0/267 p-value= 0/00	R= 0/013 p-value= 0/837	R= 0/261 p-value= 0/00

*Model presenting*

Here, we are trying to estimate and formulate a model based on the information presented in the previous sections. To this purpose, first we estimated the applied persistent variables which are at the same level of persistency of the Dicky Fowler’s test.

Three models were estimated through the regression analysis of the Eviews software. These models are as follows:

In the first model, the short-term debt ratio to the total capital was used, and in models 2 and 3, long-term debt to the total capital and total debt to the total capital were used.

In order to make decision on the estimation of the model as pooling or panel, we used F-Limer test ,then as it was specified that the differences between companies are not meaningful so the data has been studied in a pooling manner.

## Results and Discussion

The negative relation between short-term debt ratio and the profitability in the first model means that supplying the finance through short-term debts does not lead to profitability in the companies. Moreover, the negative relation in the second model means that those companies who finance through long-term debts, face loss of profitability.

**Table 3:** Estimation results of regression models

**D.W**	**F-statistic**	**R** ^2^	**p-value**	***β***	**X**	**NIS**
1.90	336.89 Prob: 0.000	0/797	0/000	-1.055	C	Model 1
			0/049	-0/036	STDTA	
			0/000	0.254	SF	
2.26	543.18 Prob: 0.000	0/796	0/000	-0/811	C	Model 2
			0/000	-2/817	LTDTA	
			0/000	0/220	SF	
1.90	333.473 Prob: 0.000	0/770	0/000	-1/026	C	Model 3
			0/011	-0/046	TDTA	
			0/000	0/250	SF	

The negative and meaningful relationship between profitability and capital structure in all the three models, is in line with the pecking order theory and information asymmetrical theory and is compatible with the findings of studies done by Rajan and Zeegnals (1995), Fama and Frech (1998), Yas *et al*. (2001), Chen (2004), Lara and Meskoeeta (2003) and Hang and Sung (2006).

In conclusion, it is worth mentioning that in Iran’s pharmaceutical industry during years 2001-2010, the capital structure of the companies has been based on pecking order theory, which means that companies prefer to supply finance internally, rather than outsourcing; they also prefer financing through borrowing to financing through issuing stocks.

It can be concluded that managers and decision makers are forced to use internal financing since outsourcing may cause some information to be released to the outside investors, leading to the company to be probably under the control of the outside investors. So they prefer inside financing comparing to the outside one.
